# Diminishing Availability of Publicly Funded Slots for Antiretroviral Initiation among HIV-Infected ART-Eligible Patients in Uganda

**DOI:** 10.1371/journal.pone.0014098

**Published:** 2010-11-24

**Authors:** Elvin H. Geng, Mwebesa B. Bwana, Jerome Kabakyenga, Winnie Muyindike, Nneka I. Emenyonu, Nicholas Musinguzi, Peter Mugyenyi, Jeffrey N. Martin, David R. Bangsberg

**Affiliations:** 1 Division of HIV/AIDS, Department of Medicine, San Francisco General Hospital, San Francisco, California, United States of America; 2 Department of Epidemiology and Biostatistics, University of California San Francisco, San Francisco, California, United States of America; 3 Ragon Institute of Massachusetts General Hospital, Massachusetts Institute of Technology and Harvard, Massachusetts General Hospital Center for Global Health, Harvard Medical School, Boston, Massachusetts, United States of America; 4 Mbarara University of Science and Technology, Mbarara, Uganda; 5 The East Africa International Databases to Evaluate AIDS Consortium; University of Cape Town, South Africa

## Abstract

**Background:**

The impact of flat-line funding in the global scale up of antiretroviral therapy (ART) for HIV-infected patients in Africa has not yet been well described.

**Methods:**

We evaluated ART-eligible patients and patients starting ART at a prototypical scale up ART clinic in Mbarara, Uganda between April 1, 2009 and May 14, 2010 where four stakeholders sponsor treatment – two PEPFAR implementing organizations, the Ugandan Ministry of Health – Global Fund (MOH-GF) and a private foundation named the Family Treatment Fund (FTF). We assessed temporal trends in the number of eligible patients, the number starting ART and tabulated the distribution of the stakeholders supporting ART initiation by month and quartile of time during this interval. We used survival analyses to assess changes in the rate of ART initiation over calendar time.

**Findings:**

A total of 1309 patients who were eligible for ART made visits over the 14 month period of the study and of these 819 started ART. The median number of ART eligible patients each month was 88 (IQR: 74 to 115). By quartile of calendar time, PEPFAR and MOH sponsored 290, 192, 180, and 49 ART initiations whereas the FTF started 1, 2, 1 and 104 patients respectively. By May of 2010 (the last calendar month of observation) FTF sponsored 88% of all ART initiations. Becoming eligible for ART in the 3^rd^ (HR = 0.58, 95% 0.45–0.74) and 4^th^ quartiles (HR = 0.49, 95% CI: 0.36–0.65) was associated with delay in ART initiation compared to the first quartile in multivariable analyses.

**Interpretation:**

During a period of flat line funding from multinational donors for ART programs, reductions in the number of ART initiations by public programs (i.e., PEPFAR and MOH-GF) and delays in ART initiation became apparent at the a large prototypical scale-up ART clinic in Uganda.

## Introduction

Although multinational HIV/AIDS treatment programs have been one of greatest public health successes this century, diminishing resource commitments by major donors have raised uncertainty about the future of access to antiretroviral therapy (ART) and the goal of universal access. To date, the United States Congress has authorized $48 billion to combat HIV/AIDS, tuberculosis and malaria via both bilateral programs, including the President's Emergency Plan for AIDS Relief (PEPFAR), and contributions to the multilateral Global Fund to Fight AIDS, Tuberculosis and Malaria through fiscal year 2013 and have supported ART for nearly 3.7 million persons in resource limited settings [1]. Despite concerns about the feasibility of implementing life-long HIV therapy with limited health care infrastructure, the scale-up of ART has been accompanied by rates of medication adherence and viral suppression which exceeded expectations and in many cases surpassed precedents established in North America and Western Europe [2], [3], [4]. These clinical benefits have lead to evidence of increases in population level life expectancy, reduced rates of HIV/AIDS related orphans and mitigated the economic impacts of HIV/AIDS on many developing countries [5], [6]. Overall, despite the fact that early mortality [7] and losses to follow-up are high [8], the effectiveness of the global ART roll out is indisputable. These successes notwithstanding, funding at the global level has been flat during 2009–2010 [9] and the United States President's 2011 Budget Request to Congress contains 6.7 billion in total global HIV funding which represents a 2.2% increase over FY 2010 [10]. This figure is falls considerably short of anticipated needs at a time when less than 50% of persons with immediate need for ART in Africa are accessing it [11]–[14].

To date, however, relatively little public data exists that provides information on trends in the availability of slots for ART initiation and the distribution of stakeholders who sponsor ART initiation in this funding climate. The Immune Suppression Syndrome (ISS) Clinic in Mbarara, Uganda is a prototypical ART clinic and is uniquely situated to examine the relative contributions of different funders supporting ART initiations because four different stakeholders operate simultaneously in one location – two PEPFAR partner organizations, the Ugandan Ministry of Health – Global Fund and a private foundation (Family Treatment Fund; http://familytreatmentfund.org). The clinic started its first patients on ART in 2002, before large scale multilateral efforts had arrived, using funding from FTF. In 2004–2005 the clinic grew rapidly with rapid influx of PEPFAR and Global Fund support and the FTF took on an ancillary role in providing ART for patients during medication stock outs, supplying treatment for opportunistic infections and training HIV-infected patients on household income generation. We hypothesized that the recent limited increases in funding for HIV programs would translate into decreased availability of treatment slots sponsored by PEPFAR programs and place a greater reliance on programs such as FTF.

## Methods

### Ethics Statement

The study was approved by the institutional review board of University of California, San Francisco, the Mbarara University of Science and Technology and the Uganda National Counsel of Science and Technology. The ethics approvals for this study allow for the analysis of de-identified clinic data already collected in the course of routine care, therefore no specific written or verbal consent was obtained.

The ISS Clinic serves the Mbarara District (population 1.1 million, 92% in rural areas) as well as portions of adjacent districts and is a prototypical implementer of the “public health approach” to ART delivery which means that it serves a large number of patients without the benefit of sophisticated monitoring (e.g., routine HIV RNA determination) nor subspecialty trained doctors and with medications limited to “simplified and standardized” regimens in WHO guidelines [9]. As of May 14, 2010, over 18,094 patients have been seen at the ISS clinic. Four to six clinical and medical officers staff up to 250 visits a day. From the ISS Clinic we evaluated ART naïve adult patients with CD4-based indications for ART initiation and who made a visit between April 1, 2009 and May 14, 2010. Demographic and clinical characteristics were obtained from the clinic's electronic medical record system.

The stakeholder (e.g., PEPFAR implementing partner, the MOH or FTF) that sponsors ART for a new patient was abstracted from clinical records. The ART number for each patient is determined by the pharmacy staff who assigns an “ART number” prior to ART initiation. The ART number is comprised of a “prefix” that identifies the program sponsoring the ART (e.g., PEPFAR, MOH or FTF) as well as a “suffix” that uniquely identifies the patient. Once assigned, the ART number is entered into the pharmacy and clinical databases and is henceforth present on the patient's records. When the patient goes to the pharmacy to pick up medications, the ART number is used to determine the source for the prescription refill. Therefore, the ART number is a reliable link to the ART program sponsoring medications for each patient.

We calculated the number of patients starting ART each month and quartile of calendar time among eligible patients. We also tabulated the distribution of patients starting ART in each calendar month and quartile of calendar time by the organization providing funding. We used the Kaplan-Meier method to calculate time from ART eligibility (as determined by first CD4 count < or = 250 cells/cc^3^) to ART initiation stratified by calendar time to examine whether a later calendar time of eligibility was associated with a delayed ART initiation. This survival analysis was restricted to the patients who became eligible for the first time between April 1, 2009 and May 14, 2010 to avoid selecting patients with longer eligibility times into the first quartile. Calendar time was discretized into quartiles, and the effect of each quartile was compared to the first quartile as an indicator variable. The log-rank test was used to evaluate significance of observed differences. We used a Cox proportional hazards model to estimate the effect of calendar time (as partitioned in quartiles) on the rate of ART initiation adjusting for three variables of *a priori* importance (age, sex, and pre-therapy CD4 count). Pre-therapy CD4 levels were discretized as per convention at 0–50 cells/cc^3^, 51–100 cells/cc^3^, 101–150 cells/cc^3^ and 151–250 cells/cc^3^.All analyses were conducted in Stata v.10 (College Station, TX). The funders had no role in the design, analysis or interpretation of the results.

## Results

Between April 1, 2009 and May 14, 2010, 1309 patients made visits who had indications to start ART by virtue of a CD4 determination <250/cc^3^ (as per Ugandan National Antiretroviral Treatment Guidelines) [10]. As typical of HIV clinic populations in East Africa, 61% of the patients were female and 39% male. The median age was 36 year (IQR, 31–39). Median CD4+ T cell value at the time of an indication was 110 cells/cc^3^ (IQR: 39 to 175). Over 14 months, the median number of patients making visits with ART indications each month was 88 (IQR: 74 to 115). Overall the total number of patients starting ART over the last 14 months was 819. Of these 819, PEPFAR programs started 318 (39%) on ART, the MOH started 393 (48%) and FTF started 108 (13%).

**Figure 1:**
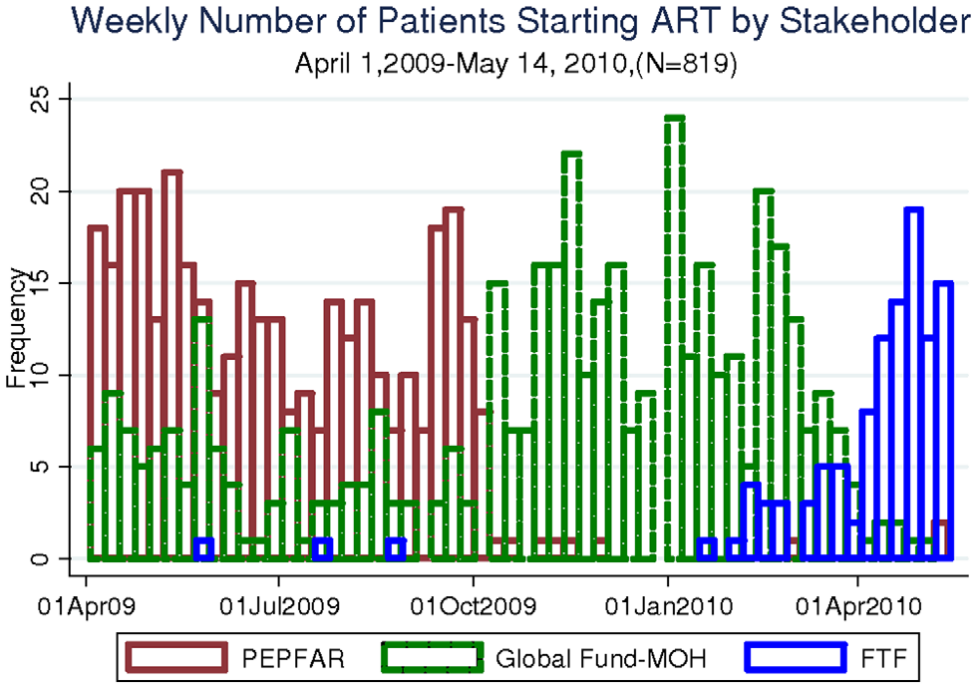
The number of ART initiations each week by stakeholder from April 1, 2009 to May 10^th^, 2010 at the ISS Clinic, Mbarara, Uganda.

**Figure 2:**
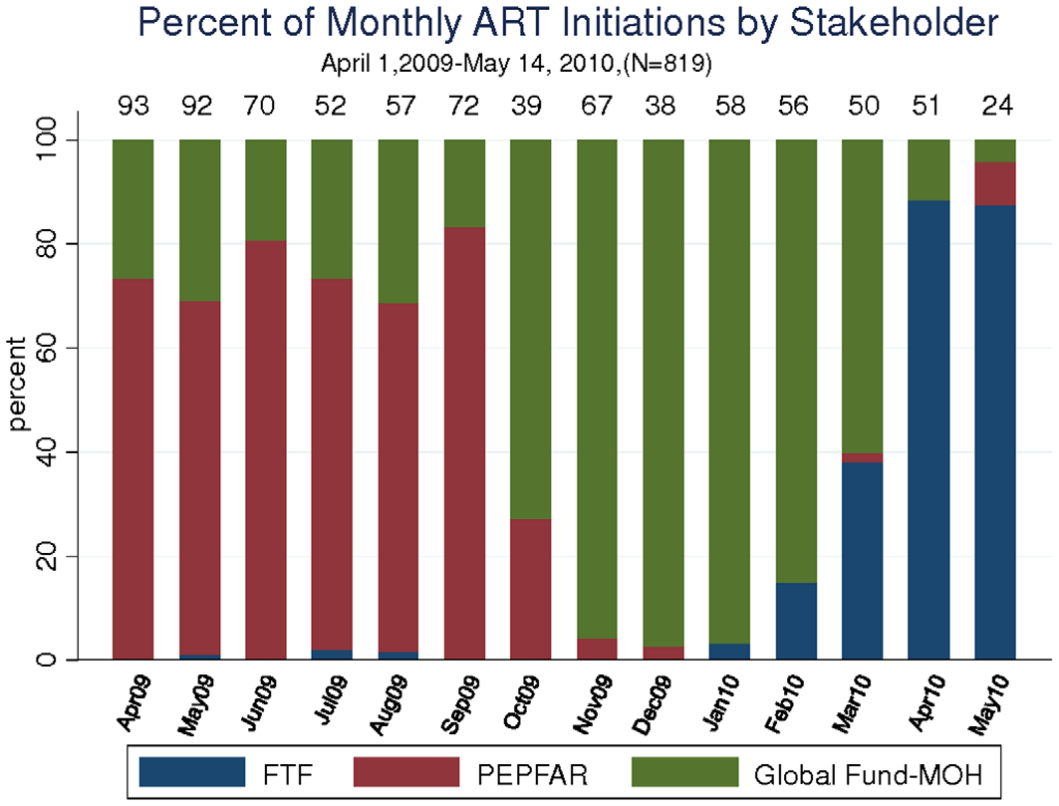
The percentage of monthly ART initiations by stakeholder, ISS Clinic, Mbarara, Uganda. The total number starting per month is shown across the top of the bars.

Secular trends show large shifts in the stakeholder providing therapy (Figure 1). Overall, the contribution of PEPFAR-sponsored ART initiations fell markedly between April 1^st^, 2009 and May 14^th^, 2010. By quartile of calendar time, PEPFAR and MOH sponsored 290, 192, 180, and 49 ART initiations whereas the FTF started 1, 2, 1 and 104 patients respectively. When examined by monthly fractions, the trends are more apparent (Figure 2). At the beginning of the interval – in April of 2009 – PEPFAR implementing partners accounted for 68/93 (73%) or ART initiations. In October PEPFAR sponsored initiations dropped rapidly to 10/39 (26%) and then continued to decline subsequently. MOH sponsored ART initiations rose in number when PEPFAR initiations declined in October 2009: while the MOH started an average of 20 patients a month between April 2009 and October 2009, by November MOH initiations rose to 61/67 (92%) of all ART initiations. MOH maintained a high proportion of starts until April and May of 2010 when MOH sponsored ART initiations then fell to 5/75 (7%). Over this 14 month interval, FTF sponsored very few ART initiations until February when FTF sponsored initiations grew to 7/57 (12%). In March FTF sponsored 18/50 (36%) ART initiations; in April FTF sponsored 45/51 (88%) initiations and at the time of the database closure on May 14^th^, FTF sponsored 21/24 (88%) initiations in May.

**Figure 3:**
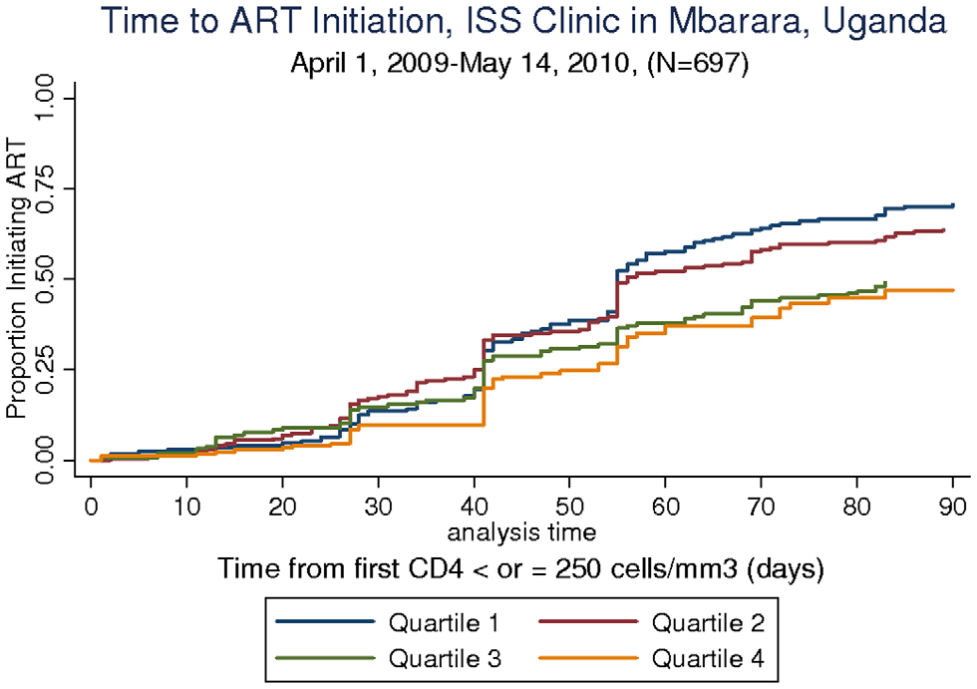
Time from eligibility for ART (as determined by Ugandan National ART Guidelines of a CD4 count less than or equal to 250 cells/mm^3^) to initiation of ART. The denominator in this analysis is restricted to the subset of patients who became eligible for ART for the first time between April 1, 2009 and May 14, 2010.

We conducted an analysis of time to ART initiation among 697 of 1309 patients who became eligible for the first time during the interval between April 1, 2009 and May 14^th^, 2010. The number who became eligible in the first through fourth quartiles respectively was: 168 (24%), 199 (29%), 156 (22%) and 174 (25%). The three month cumulative incidence of ART initiation in quartiles 1–4 respectively were 71% (95% CI: 64%–78%), 64% (95% CI: 57%–70%), 49% (42%–57%) and 47% (95% CI: 38%–57%). The log-rank test for equality of survivor functions was significant at p<0.001 (Figure 3). In a Cox model adjusting for age, sex and baseline CD4 value, the rate of ART initiation in the second quartile was 0.93 (95% CI: 0.74–1.18), in the third quartile was 0.61 (95% CI: 0.47–0.80) and in the fourth quartile was 0.53 (95% CI: 0.39–0.73) when compared to the first quartile. Age and sex were not significantly associated with the rate of ART initiation, but a baseline CD4 value of 0–50 cells/cc^3^ was associated with an increased rate of ART initiation (HR = 1.43, 95% CI: 1.12–1.83) compared to the reference group of baseline CD4 from 150–250 cells/cc^3^ (Table 1).

**Table 1:** Cox proportional hazards model on the association of calendar time of ART eligibility (as determined by CD4 level) and rate of ART initiation, adjusting for age, sex and pre-therapy CD4 value.

Factor	Hazard ratio	95% Conf. Interval	P-value
Calendar time			
Quartile 1 (Apr.1, 2009-Jul.15, 2009)	ref		
Quartile 2 (Jul.16, 2009-Nov.30, 2009)	0.93	0.74–1.18	0.57
Quartile 3 (Dec.1, 2009-Feb.15, 2010)	0.61	0.47–0.80	<0.01
Quartile 4 (Feb.16, 2010-May 14, 2010)	0.53	0.39–0.73	<0.01
Age	1.00	0.99–1.01	0.65
Sex	1.03	0.83–1.26	0.80
Pre-therapy (i.e., baseline) CD4 value			
0–50 cells/mm^3^	ref		
51–100 cells/mm^3^	1.18	0.81–1.55	0.65
101–150 cells/mm^3^	1.05	0.79–1.41	0.45
151–250 cells/mm^3^	1.43	1.21–1.83	<0.01

## Discussion

We found diminishing ARV supply for new ART initiation by large PEPFAR sources and an increasing reliance on FTF at a prototypical HIV clinic in southwestern Uganda during a period when ART-eligible patients continued to present to care. We also found a nearly 50% decline in the rate of ART initiation for patients who presented in the final quartile of calendar time (from February to Mary 2010) as compared to the first quartile (April to mid-June, 2009) that translates into a decrease in the 90 day cumulative incidence of ART initiation from 71% to 47% or an absolute difference of 24%. These delays in ART initiation at the ISS Clinic during this period of time coincided with a rapid fall in the number of slots available from PEPFAR in October of 2009 –the time of the “PEPFAR Halt Memo” which is a widely circulated document putatively from US CDC in Uganda requesting implementing partners stop routine initiation of new patients on ART [11]. Given fluctuations in the delivery of new treatment slots from MOH, ART initiation at the ISS Clinic during March to May 2010 was largely covered by a small foundation – the Family Treatment Fund. With an average of 88 patients a month who present with indications for ART and the fact that FTF is rapidly reaching capacity, access to ART for the steady stream of eligible patients may continue to be less timely and reliable in the foreseeable future without sustained coverage from the major stakeholders.

These data support concerns raised in the popular press and by advocacy groups that flat line funding will lead to a step backwards in the fight against HIV/AIDS. Groups like Doctors without Borders have made high profile lobbying efforts in the United States about the consequences of limiting or delaying ART initiation and have warned of the return of waiting lists [12]. Indeed, for those with CD4 levels of <200 cells/cc^3^, mortality in Africa without ART reaches 30% by 1 year and therefore even incremental delays in ART initiation are likely to lead to increases in preventable HIV-related deaths. Multiple funders have, we believe, relatively insulated the ISS Clinic from individual stakeholder shortfalls. This is not the case in smaller clinics in the community, where limited ART initiations by one stakeholder are not so easily accommodated. The impact of PEPFAR funding limitations on the number of and time to ART initiation in eligible patients may be more pronounced at those sites.

While we observed dramatic shifts in funding source at a single clinic, we recognize that the absolute level of funding at the global or national level is not the only determinant of ART availability in sub-Saharan Africa. Consistent ART supply requires procurement, storage and delivery of medications [13]. At the recent 2010 International AIDS Society Conference in Vienna, a number of global health leaders called for maximizing the availability of ART through exploiting “efficiencies”[14] in delivery of HIV/AIDS care. Further research on maximizing efficiencies is necessary to increase capacity to meet projected geometric increases in demand in setting of linear increases in funding [13].

This study has several limitations. First, while we describe rapid changes in treatment source concurrent with changes in funding allocation we cannot exclude important unmeasured factors that determine ART delivery. These factors may include the mechanisms in the delivery of ART supplies, the pharmacy stock available at the Ugandan national level among others. However, our findings of a rapid decline in PEPFAR-supported ART initiations in November 2009 coincides with a October 29, 2009 letter from CDC Uganda instructing PEPFAR implementing partners to cease routine enrollment of new ART patients [12] which supports a causal interpretation. A second limitation is that the center which we studied has four simultaneous stake holding organizations (e.g., two PEPFAR implementing partners, the MOH and FTF). While this unusual arrangement limits the generalizability of our findings to other ART sites in the region, it provides a unique opportunity to examine trends across all stakeholders and therefore act as a gauge of the ART availability in the region. Based on anecdotal evidence, the restrictions in ART initiations for in lower-level health centers surrounding the Mbarara ISS Clinic in southwestern Uganda were more – not less – severe.

In summary, these findings suggest that changes in the multinational funding landscape had an impact on ART initiation at the clinic level in Uganda. This effect occurred via a sudden reduction in the number of new PEPFAR-funded ART treatment initiations and a letter from US CDC instructing implementing partners to stop enrolling new patients [12]. If these trends continue, increased efficiency of care delivery may not be sufficient to meet projected increases in demand for treatment. Couples, friends and family already on ART may experience pressure to share ART medications with loved ones who are ART eligible but on waiting lists, leading to partial viral suppression, increased drug resistance, and thereby impairing future ART treatment benefits once additional resources become available. ART initiation declines may also reverse favorable trends in HIV disease prevention efforts that include reduction of stigma, rise in acceptability of testing and penetration of public health messages into society. Increasing commitments from both multilateral donors and national governments in Africa will be required to secure the achievements to date.
